# The stigma-induced diagnostic gap: a critical barrier to leprosy elimination^؆^

**DOI:** 10.1016/j.abd.2026.501458

**Published:** 2026-08-29

**Authors:** David Ramírez Portilla

**Affiliations:** School of Health Sciences, Universidad Nacional Abierta y a Distancia, Bucaramanga, Colombia

Dear Editor,

The findings by Cruz et al.^1^ regarding the pervasive nature of leprosy-related stigma in Brazil are more than a psychosocial observation; they reveal a critical blind spot in epidemiological surveillance. Their study showed that stigma persists among patients, contacts, and the general population, with shame, negative self-opinion, and social avoidance emerging as central elements across groups. These findings suggest that stigma may interfere with early consultation, disclosure, contact tracing, and treatment adherence.

Current technical guidance, such as the recent practical review by Fróes Júnior et al.,^2^ provides valuable frameworks for clinical and laboratory diagnosis, including histopathology, RLEP-PCR, anti-PGL-1 serology, monofilament testing, and ultrasonography. However, even the most accurate diagnostic tools may have limited public health impact when affected individuals delay or avoid care because of fear, shame, or anticipated discrimination.

We therefore propose the concept of a stigma-induced diagnostic gap: the distance between diagnostic capacity and real-world access to diagnosis created by social concealment and avoidance. In endemic regions, stigma may contribute to hidden prevalence by keeping symptomatic individuals outside the reach of early detection and by weakening contact surveillance.

“Zero stigma” should not be regarded only as an aspirational target for 2030 but as a clinical and programmatic requirement today.^3^ Dermatology services in endemic areas should consider incorporating validated stigma screening tools into routine leprosy care, alongside neurological assessment, disability grading, and bacteriological evaluation.

If leprosy programs continue to treat *M. leprae* while underestimating social isolation, elimination efforts will remain incomplete. Closing the stigma-induced diagnostic gap is essential to transform biomedical cure into true public health control.

## Declaration of generative artificial intelligence (AI)

Generative artificial intelligence was used only for language editing and manuscript structuring. The author reviewed and approved the final content and takes full responsibility for the manuscript.

## Financial support

None declared.

## Authors' contributions

David Ramírez Portilla: Conception and design of the manuscript; Critical review of the literature; Drafting and editing of the manuscript; Critical review of important intellectual content; Approval of the final version of the manuscript.

## Research data availability

Does not apply.

## Conflicts of interest

None declared.

## References

[1] Cruz P.T., Miot H.A., Talhari C., Pedrosa V.L., Serique R.M., Cordeiro A.G.S. (2026). Stigma associated with leprosy among patients, contacts, and the general population in an endemic region of Brazil. An Bras Dermatol..

[2] Fróes Júnior L.A.R., Sotto M.N., Avancini J., Garbino J.A., Trindade M.ÂB. (2026). Laboratory and functional tests in leprosy diagnosis: a practical guide for clinical decision-making. An Bras Dermatol..

[3] World Health Organization (2021). Towards zero leprosy: global leprosy (Hansen’s disease) strategy 2021-2030 [Internet].

